# P-915. Risk of Psychiatric Disorders Associated with Doxycycline Use Compared to Other Antibiotics: A Large-Scale Real-World Data Analysis

**DOI:** 10.1093/ofid/ofaf695.1121

**Published:** 2026-01-11

**Authors:** Kausik Maiti, Aleksandar Urosevic, Anne Kasmar

**Affiliations:** Parexel International, Hyderabad, Telangana, India; Parexel International, Hyderabad, Telangana, India

## Abstract

**Background:**

Doxycycline is a widely used semisynthetic tetracycline antibiotic recognized for its broad-spectrum antimicrobial activity. Recently, the U.S. Food and Drug Administration (FDA) announced that it is evaluating the need for regulatory action regarding the safety signal of psychiatric disorders associated with doxycycline use. This study aims to investigate the association between doxycycline and other commonly prescribed antibiotics with the occurrence of psychiatric disorders using large-scale real-world data (RWD)

**Methods:**

This retrospective observational study utilized the TriNetX global dataset (226,225,538 patients). Six cohorts were created comprising patients who received doxycycline, clarithromycin, amoxicillin, sulfamethoxazole, ciprofloxacin, and cephalosporins (1st-3rd generation). Patients with pre-existing substance-induced mental disorders were excluded. The risk of developing mood disorders (ICD-10-CM: F30-F39), anxiety disorders (F40-F48), and psychotic disorders (F20-F29) within 90 days of antibiotic initiation was compared between doxycycline and other antibiotic cohorts using Measure of Association Analysis

**Results:**

Doxycycline use was associated with a higher risk of psychiatric disorders compared to other antibiotics. For mood disorders, doxycycline showed increased risks ranging from 0.5 to 2.5%. For anxiety disorders, the increased risks ranged from 0.9 to 3.0%. The risk differences for psychotic disorders were smaller, with doxycycline showing slightly higher risks (0.1%) compared to some antibiotics, and negligible or slightly lower risks compared to others

**Conclusion:**

This RWD analysis demonstrates a consistent association between doxycycline use and a small increased risk of psychiatric disorders, particularly mood and anxiety disorders, compared to other antibiotics. These were statistically significant (p< 0.001), likely due to large sample sizes, although the clinical significance of these differences requires careful interpretation given the small absolute risk differences. These findings align with the FDA's ongoing evaluation and contribute valuable evidence to inform clinical decision-making. Further research is needed to elucidate underlying mechanisms and identify high-risk patient subgroups.

**Disclosures:**

Anne Kasmar, Eli Lilly and Company: Stocks/Bonds (Public Company)|Novo Nordisk: Stocks/Bonds (Public Company)

